# Bolstering human rabies surveillance in Africa is crucial to eliminating canine-mediated rabies

**DOI:** 10.1371/journal.pntd.0006367

**Published:** 2018-09-06

**Authors:** Anaïs Broban, Mathurin C. Tejiokem, Issaka Tiembré, Sophie Druelles, Maïna L’Azou

**Affiliations:** 1 Global Epidemiology, Sanofi Pasteur, Istanbul, Turkey; 2 Pasteur Institute of Cameroon, Department of Epidemiology and Public Health, Yaoundé, Cameroon; 3 Anti-Rabies Center, National Institute of Public Hygiene, Abidjan, Ivory Coast; 4 Global Epidemiology, Sanofi Pasteur, Lyon, France; Ross University School of Veterinary Medicine, SAINT KITTS AND NEVIS

Canine rabies has been controlled or eliminated in most developed countries through mass dog vaccination programs. However, the transmission of rabies to humans continues to be a formidable public health problem in many developing countries, where unvaccinated dogs continue to be a substantial reservoir of human disease. Globally, rabies causes an estimated 59,000 human deaths each year, particularly in Asia (approximately 35,000 deaths) and Africa (21,000−25,000 deaths) [1,2]. However, these estimates are derived from projected dog bite incidence rates and other estimated factors for individual countries, and though they are many times higher than the rabies incidence rates reported by the national authorities of these countries, they likely do not accurately reflect the true burdens of the disease [3]. Together with a lack of disease awareness in vulnerable communities (particularly rural and impoverished ones) and a lack of political will to address rabies control, unreliable surveillance data contributes to a cycle of neglect in countries where the disease burden is highest [1,4,5].

Yet the means exist to bring rabies under control in countries where it continues to be endemic. Recognizing this, the World Health Organization (WHO), the World Organization for Animal Health (OIE), the Food and Agriculture Organization of the United Nations (FAO), and the Global Alliance for Rabies Control (GARC) have proposed a feasible, strategic global plan to end canine-mediated human rabies by 2030 [6–8].

Examples of successful rabies control can be found in many parts of Latin America, where large-scale, nationally mandated dog vaccination campaigns in most countries across the region have greatly reduced the numbers of dog cases, human cases, and human deaths [9]. Sporadic vaccination campaigns began in the 1970s in the larger cities in Latin America, for example, with over 200,000 dogs vaccinated in Mexico in 1970 [10]. Yet even as vaccination programs continued to gradually increase in the country, with over 1 million vaccine doses/year administered in Mexico by 1980, they remained insufficient because dog and human rabies case numbers continued to occur at much the same pace in the 1970s and 1980s. Two political developments can be linked to the beginning of measurable progress in rabies control: the recognition that rabies is a public health problem, rather than an agricultural one, which resulted in sustained national budgets for rabies control and the collective pledge made by many Latin American countries in 1983 and supported by the Pan American Health Organization to eliminate human rabies by 2005 [11]. With these international commitments and sustained mass dog vaccination programs with newly developed inactivated cell-culture rabies vaccines having improved immunogenicity and availability, human rabies cases eventually began to decline. Though the 2005 goal was not met, human rabies case numbers throughout Latin America decreased by 99% over this period, from 300 in 1980, 270 in 1990, 39 in 2002, to a low of three cases in 2015 [9–11]. From these experiences, it is clear that even with coordinated efforts and political support, regional rabies control can take decades of sustained effort to achieve. In a specific example, it took Mexico over 20 years of progressively increasing nationwide dog vaccination efforts, from 5 million doses per year in 1989 to 15 million doses per year delivered since 2005 to achieve zero human rabies deaths in 2015 [10]. Nevertheless, with the lessons learned from these successful long-term efforts and implementable strategies for regional control, along with the current availability of potent vaccines, we may have the means to control rabies in Africa more quickly than in Latin America [12–14].

However, compared to the Latin American commitments made in the early 1980s, African countries have yet to achieve the collective international recognition of rabies as an urgent public health problem, so the cycle of neglect persists in many countries. The principal reason for this is that the extent of the disease burden there is not well understood or characterized due to inadequate surveillance. To break the cycle of neglect, robust surveillance is needed to obtain the accurate data necessary to raise awareness of rabies in the population, to engage the community, and to create the political will and intersectoral collaboration needed to initiate changes in rabies policies [12,15,16]. Ongoing regional disease incidence estimates also allow control measures to be deployed preferentially in areas at highest risk or in case of outbreaks and provide a baseline against which the impact of subsequent rabies control programs can be measured over time.

Unfortunately, rabies surveillance throughout most of Africa is weak. In a recent global survey of surveillance systems in rabies-endemic countries, of which 49 of the 54 African countries are considered as moderate- to high-risk categories for human rabies, surveillance systems were deemed ineffective in at least 16 of the 23 that responded to the survey [17]. Though animal bites and cases of animal and human rabies are currently notifiable events in most African countries, poor or nonexistent surveillance render national epidemiological data unreliable due to substantial under-reporting [5,17]. Surveillance systems are usually passive, based on clinical symptoms, and capture few authentic human cases due to inaccurate diagnosis and the scarcity of laboratory confirmation [5,18]. In addition, bite victims may not seek standard medical treatment due to ignorance, poor availability, excessive travel distance from remote communities, and high cost [5]. Treatment, especially in rural areas, may also be sought from traditional healers who are outside the healthcare system and do not report bites or rabies cases [19–22]. In addition, healthcare providers (HCPs) may lack the training needed to deliver appropriate case management and may not recognize rabies signs and symptoms once the disease develops in humans, misdiagnosing it as cerebral malaria or another disease [18]. Even if the disease is correctly diagnosed by clinical criteria, very few cases are submitted for laboratory confirmation. Further, in the absence of any treatment options once the disease has taken hold, patients may return home to die, in which case neither their death nor its cause are recorded in the health or surveillance system [1,17,23]. With surveillance in many countries poorly equipped to detect and confirm human rabies cases, many authentic infections are not recorded, and the data are unavailable to support efforts to control the disease.

The poorly defined epidemiology of rabies stalls political support for its control. Indeed, control may be considered infeasible by national authorities due to uncertainties about wildlife reservoirs of rabies viruses, the seemingly overwhelming task and cost of vaccinating large numbers of domestic and stray dogs, and the level of canine vaccination coverage needed to control the disease [13,24–26]. Thus, rabies control and surveillance are given a low priority against competing public health concerns. Existing regulations for reporting the disease may be poorly enforced and international guidelines poorly implemented. With limited resources and funding, only a small portion of cases make their way up the chain through the surveillance system to be captured in the national registry.

To reverse this neglect, effective surveillance systems are needed. However, implementing an active surveillance system can be a challenge, especially in rural areas of developing countries—precisely where they are needed most. Fortunately, guidelines and surveillance standards, such as the Rabies Surveillance Blueprint, which describes important features of quality rabies surveillance programs, are available [27,28]. Effective surveillance consists of several key concepts. Rabies must first be a legally notifiable disease. HCPs must be engaged and trained in surveillance methods, disease diagnosis, and case recognition according to internationally recognized definitions. Community participation is also a key component. Local populations at risk must be made aware of the disease and its symptoms in humans and animals to increase and encourage the reporting of suspected cases and suspected rabid animals and to ensure that animal exposure victims seek immediate appropriate medical treatment and postexposure prophylaxis (PEP), if warranted. Laboratory capabilities are also necessary for confirming suspected animal and human cases, and if not available locally, quality storage and shipping supplies are needed to transport samples from remote areas to central laboratories [29]. The data collected on suspected human rabies cases, human exposures, and rabid animals must be analyzed and disseminated effectively. Communication among the various national levels of healthcare administration is a key means of disseminating results. Reporting networks need to be efficient and timely so that a high proportion of the suspected and confirmed cases and animal exposures captured by the system are communicated throughout the network to enable control efforts to be allocated appropriately in the regions of the highest risk. Communication and coordination between human health and veterinary systems are also crucial for the follow up of both human and animal cases. Finally, stakeholders need to be engaged long term to ensure that the surveillance is sustainable.

Rabies has been a widespread public health problem in Africa for decades, and a number of organizations, including the African Rabies Expert Bureau (AfroREB), the Southern and Eastern African Rabies Group (SEARG), the Rabies in West Africa group (RIWA), the Global Alliance for Rabies Control (GARC), and the Pan-African Rabies Control Network (PARACON), have been engaging people in the fight against rabies and promoting rabies surveillance and control over the past decade [16]. In addition to guidance provided by the Rabies Surveillance Blueprint, the Stepwise Approach to Rabies Elimination (SARE) describes five stages of measurable progress toward rabies control and elimination. This tool is an instrument that countries can use to perform regular progress assessments. Most African countries are in the early stages of SARE (stage 1 or 2), in which rabies has been confirmed and reported to global organizations (e.g., WHO), surveillance systems are beginning and progressing, rabies action plans are in development, and the availabilities of canine vaccination and PEP are increasing [30]. As a recent example, application of the SARE tool at a national meeting in Ethiopia identified a number of important gaps where national and regional efforts need to be prioritized, namely intersectoral communication and dog vaccine availability [31]. In contrast, their existing surveillance system was identified as a strength. Addressing these critical gaps will accelerate the development of an effective rabies control strategy and enable Ethiopia to progress to SARE stage 1. However, some countries have made little or no progress (stage 0), and few countries have progressed to stage 3, further indicating that surveillance programs in most countries are incomplete at best and need to be improved.

Strengthening existing surveillance systems is an implementable action in Africa. In two accompanying reports, we describe efforts to strengthen regional surveillance programs in Cameroon and Ivory Coast, together with the results of over two years of surveillance [32][33]. The programs focused on engaging HCPs and veterinary authorities and improving their training in rabies surveillance, animal bite management, human rabies case diagnosis and management, as well as sampling techniques and sample management. Materials were provided for sample storage and shipment to increase the proportion of suspected cases that were sampled and submitted for laboratory confirmation. The reporting arm of the programs included detailed case report forms that were collected, analyzed, and shared through a network of partnerships established between local health centers, regional health units, the Pasteur Institute in each country, and the national public health ministry. Public awareness programs were also implemented among healthcare and veterinary professionals and communities through leaflets, advertisements, media announcements, and participation in World Rabies Days.

The surveillance results obtained in each country suggest that animal exposures and rabies cases had been under-reported by national systems, confirming that improved surveillance is needed to more accurately estimate the burden of rabies in these countries, as in others. In the West Region of Cameroon, 1,402 exposures were reported along with four suspected human cases, one of which was laboratory confirmed. In 2014 alone, a total of 507 exposures were reported for all 20 districts in the West Region, 480 of which were captured in the 11 districts participating in the enhanced surveillance project [33]. This is nearly nine-fold more than the 57 exposures reported for the entire West Region in 2013 [34]. In Ivory Coast, 31,492 animal exposures and 50 suspected human cases were reported, and 32 of the 39 samples submitted for testing were laboratory confirmed [32]. By comparison, 26 cases of human rabies for the entire country were reported to the Ivory Coast national health ministry between 2001 and 2009, only four of which were sampled and then confirmed by postmortem laboratory analysis [35].

These two strengthening programs demonstrate that it is possible to reinforce the surveillance capacity in these countries and motivate the actors involved at all levels. However, some remaining issues need to be resolved so that these surveillance systems become sustainable over time, which will be critical for identifying and understanding changing trends and patterns in rabies transmission and for assessing the impact of future rabies control efforts. Clearly, one challenge will be to secure long-term financing of these programs and their expansion across other regions in the countries. Both of these pilot programs were subsidized and carried out in collaboration with Sanofi Pasteur and show that partnerships with private enterprises can be an effective means to obtain the initial data critical for leveraging long-term governmental support for rabies control. Efforts to obtain this support are in progress.

Despite the documented increases in exposures and human rabies cases compared to previous years, projections of these results across the respective national populations yielded incidence estimates that are still far below those previously made for Cameroon and Ivory Coast using comprehensive models [2]. We suspect that many at-risk exposures were not captured by surveillance because these individuals did not seek medical treatment at all or, in the case of Ivory Coast, they may have received initial treatment at a local health center but did not seek PEP or additional follow-up treatment at a National Institute of Public Health center where the surveillance system began. Surveillance in both countries could be made more efficient by raising rabies awareness in local and rural communities so that people understand the critical need to seek immediate medical treatment after an at-risk animal exposure and to complete PEP treatment once it has begun. In this way, exposure victims would be more likely to enter the healthcare system and be recorded by surveillance. Surveillance could also be enhanced by conducting follow-up investigations of animal exposures. In Haiti, systematic follow ups to animal exposures revealed additional victims who had not sought medical care, many of whom should have received PEP [36]. Unfortunately, the human resources to systematically investigate exposures are not yet available in either country, though veterinarians in Cameroon are available to investigate a limited number of reports of suspected rabid animals.

Other areas identified for improvement include expanding the sampling of suspected animal and human cases and attaining the complete and sustained collaborative engagement of all partners (HCPs, veterinary and human health authorities). In each program, samples were not obtained and submitted for laboratory testing in all cases, preventing the systematic confirmation of suspected animal and human cases. Such evidence is needed to unequivocally demonstrate the extent to which rabies is present. In Ivory Coast, sampling of human cases was quite efficient, as 64% of the 50 suspected cases were laboratory confirmed, and these provided quality rabies data. In Cameroon, however, only one of the four suspected human cases was laboratory confirmed. Additional rabies confirmations could have been obtained from animal cases, but only 14% of the human exposure cases were followed up with a veterinary investigation, and only four of seven animal samples submitted for testing were confirmed positive. Expanded sampling of human and animal cases in Cameroon will be needed to better demonstrate the presence of rabies.

Nevertheless, these results represent major improvements in the implementation of routine testing and confirmation, since prior to the reinforcements, only very few human samples in Ivory Coast and no samples in Cameroon were collected or tested. Training of healthcare and veterinary professionals must continue so that local actors understand the crucial importance of their efforts, and this can be further reinforced through retro-information and feedback on the performance of the surveillance. Specific and timely retro-information also provides an essential tool for disseminating accurate disease information throughout local communities to increase public awareness on the presence and prevention of rabies as well as on the importance and effectiveness of PEP. Additionally, information about local beliefs and practices should be collected to identify knowledge gaps in the community that may affect rabies control strategies. For example, dog meat is considered a delicacy in Cameroon and the hazards associated with the butchering and handling of potentially rabid animals need to be communicated throughout local communities. Local preferences to seek treatment from traditional healers must also be addressed. In several surveys in Ethiopia, over 50% of the individuals surveyed believed that traditional healers were able to treat or cure rabies in humans and animals [20–22]. Thus, campaigns to persuade exposure victims to seek medical care rather than inappropriate treatment from traditional healers could improve surveillance efficiency and PEP uptake and reduce rabies cases. Finally, it may be necessary to create a specific government unit in the health ministry of each country to manage surveillance across public health and veterinary services. In a successful rabies elimination demonstration project in Tanzania, this capacity was identified as a critical need in the development of their roadmap for disease elimination [37].

To meet the goal of eliminating dog-mediated human rabies deaths by 2030 in Africa and elsewhere, accurate active surveillance will be crucial for ending entrenched rabies neglect and engaging the resources necessary to control it. The subsidized programs in Cameroon and Ivory Coast demonstrate that pre-existing but poorly functioning surveillance systems can be improved regionally and nationally, with limited financial support, to provide data that can be used to better estimate the burden of rabies. With the improved quality of the reported burdens of disease, decision-makers may now have sufficient information to be convinced that rabies elimination programs are needed in these countries and that model systems are in place to assess the impact of control efforts.

The programs also provide realistic and implementable models that can be shared with other countries seeking to improve surveillance. The data collected can be used to inform the PARACON web-based surveillance bulletin, an online data repository that allows automatic data sharing among sub-Saharan African countries [38]. As robust rabies surveillance and the hard evidence for the extent of the disease that it provides are both needed throughout Africa, such international strengthening initiatives will be instrumental to garner the attention and support of health ministries, local communities, and other stakeholders to address the problem and break the cycle of neglect.
